# Citrate synthase lysine K215 hypoacetylation contributes to microglial citrate accumulation and pro‐inflammatory functions after traumatic brain injury

**DOI:** 10.1111/cns.14567

**Published:** 2024-02-08

**Authors:** Fengchen Zhang, Tao Lv, Jie Li, Jie Lian, Hui Wu, Yichao Jin, Feng Jia, Xiaohua Zhang

**Affiliations:** ^1^ Department of Neurosurgery Ren Ji Hospital, Shanghai Jiao Tong University School of Medicine Shanghai China; ^2^ Department of Neurosurgery Nantong First People's Hospital, Affiliated Hospital 2 of Nantong University Nantong China

**Keywords:** acetylation, citrate synthase, microglia, neuroinflammation, TCA cycle, traumatic brain injury

## Abstract

**Aims:**

This study aimed to investigate the relationship between microglial metabolism and neuroinflammation by examining the impact of citrate accumulation in microglia and its potential regulation through Cs K215 hypoacetylation.

**Methods:**

Experimental approaches included assessing Cs enzyme activity through Cs K215Q mutation and investigating the inhibitory effects of hesperidin, a natural flavanone glycoside, on citrate synthase. Microglial phagocytosis and expression of pro‐inflammatory cytokines were also examined in relation to Cs K215Q mutation and hesperidin treatment.

**Results:**

Cs K215Q mutation and hesperidin exhibited significant inhibitory effects on Cs enzyme activity, microglial citrate accumulation, phagocytosis, and pro‐inflammatory cytokine expression. Interestingly, Sirt3 knockdown aggravated microglial pro‐inflammatory functions during neuroinflammation, despite its proven role in Cs deacetylation.

**Conclusion:**

Cs K215Q mutation and hesperidin effectively inhibited microglial pro‐inflammatory functions without reversing the metabolic reprogramming. These findings suggest that targeting Cs K215 hypoacetylation and utilizing hesperidin may hold promise for modulating neuroinflammation in microglia.

## Graphical abstract

Microglial metabolism and neuroinflammation are linked. Hypoacetylation of Cs K215 enhances microglial enzyme activity and citrate accumulation. Cs K215Q and hesperidin inhibit microglial Cs activity and pro‐inflammatory functions without reversing metabolic reprogramming. Sirt3 deacetylation of Cs is involved, but Sirt3 knockdown worsens microglial pro‐inflammatory functions.

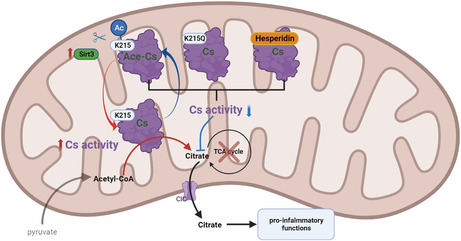

## 1 INTRODUCTION

Microglia are the most important resident immune cells in the central nervous system and play key roles in immune surveillance and neuroinflammatory pathological processes.^1^ In recent years, the link between microglial immune function and its metabolic state has received further attention. Metabolic pathways involved in microglial activity adapt to and contribute to the microglial activation phenotype. It has been well‐studied that microglia tend to switch from the oxidative phosphorylation (OXPHOS) to the aerobic glycolytic pathway when activated during brain inflammation, which is similar to Warburg effect in tumor cells^2^, ^3^ and called metabolic reprogramming.

Citrate is produced from the aldol condensation of oxaloacetate and acetyl‐CoA in the citric acid cycle (also known as the tricarboxylic acid cycle, TCA cycle). This reaction is catalyzed by citrate synthase (CS). In TCA cycle, citrate is converted into isocitrate via cis‐aconitate by the enzyme aconitase.^4^ In pro‐inflammatory microglia, citrate accumulates and is transported into the cytoplasm by mitochondrial citrate carrier (CIC), which may be due to the disruption of TCA cycle and increased glycolysis flow.^3^ In human macrophages, mitochondrial citrate export and breakdown is associated with the production of several important pro‐inflammatory mediators, like NO, ROS, and prostaglandin E2 (PGE2), etc.^5^, ^6^, ^7^ Citrate in cytoplasm could be further cleaved into oxaloacetate and acetyl CoA. Acetyl CoA in cytoplasm could enter fatty acid synthesis and provide necessary substrates for the acetylation modification of various proteins.^7^, ^8^ Overall, increased level of citrate is an important feature of pro‐inflammatory activation of microglia and macrophages.

Posttranslational modification (PTM), including phosphorylation, acetylation, methylation, etc., is one of the most effective ways of protein modification. PTMs are commonly used to control metabolic flux because the time scale of gene expression is too long to balance the rapid turnover of metabolites.^9^ In some recent studies, lysine acetylation is regarded as a vital factor that links the metabolite acetyl‐CoA and the functions of metabolism‐related enzymes, including some key enzymes in metabolism pathways.^10^, ^11^

Hesperidin is a flavonoid commonly found in citrus fruits, particularly in the peels and membranes of oranges and lemons.^12^ Previous researches focused on hesperidin's antioxidant, anti‐inflammatory, and anticancer activities.^13^, ^14^, ^15^ Hesperidin exhibits strong antioxidant effects by neutralizing free radicals. Hesperidin also shows anti‐inflammatory properties by inhibiting inflammatory mediators and cytokines. It modulates inflammation pathways and may help diseases like arthritis.^16^ A recent study reported that hesperidin may be a potential inhibitor of Cs through molecular docking in silico.^17^ If the inhibitory effect of hesperidin on Cs could be confirmed, we can use hesperidin to study the effects of enhanced Cs activity and citrate accumulation on microglial functions during neuroinflammation.

The previous researches usually focused on how to reduce excessive pro‐inflammatory responses by reversing metabolic reprogramming of microglia and unblocking the TCA cycle and oxidative phosphorylation.^9^, ^18^ In this study, we found that the reduction of Cs acetylation modification and enhanced activity mediated by Sirt3 is an important reason for the enhanced pro‐inflammatory function of microglia. By restoring Cs acetylation levels or inhibiting Cs activity with hesperidin, the citrate accumulation and the pro‐inflammatory function of microglia can be effectively inhibited, although this strategy has little effect on restoring the microglial TCA cycle.

## 2 RESULTS

### 2.1 Increased citrate level in microglia is accompanied by significantly decreased acetylation of Cs during neuroinflammation

**FIGURE 1:**
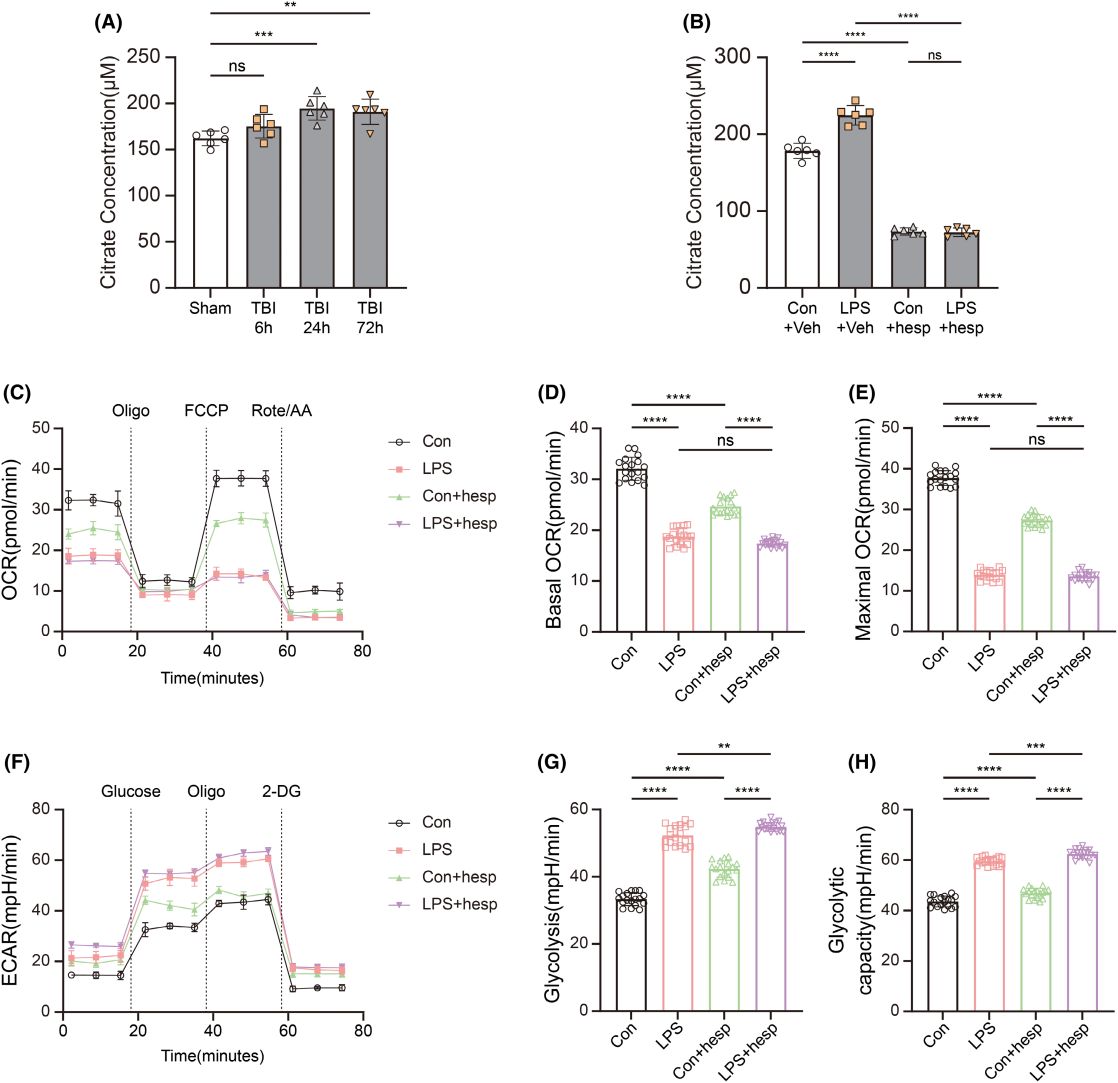
Citrate accumulation during neuroinflammation and effects of hesperidin. (A) Citrate levels in ipsilateral hippocampus 6, 24, and 72 h after traumatic brain injury (*n* = 6). One‐way ANOVA and Tukey's multiple comparisons test were used. (B) Citrate levels in primary microglia after LPS stimulation with/without hesperidin pretreatment (*n* = 6). One‐way ANOVA and Tukey's multiple comparisons test were used. (C) OCR measurements of primary microglia after LPS stimulation with/without hesperidin pretreatment (*n* = 6). (D, E) Statistics on basic OCR and maximum OCR (*n* = 6). One‐way ANOVA and Tukey's multiple comparisons test were used. (F) ECAR measurements of primary microglia after LPS stimulation with/without hesperidin pretreatment (*n* = 6). (G, H) Statistics on basic Glycolysis and Glycolytic capacity (*n* = 6). One‐way ANOVA and Tukey's multiple comparisons test were used. Bar graphs are represented as mean ± SD. ****p < 0.0001, ***p < 0.001, **p < 0.005; hesp, hesperidin; ns, no significance; veh, vehicle.

We detected the citrate level in ipsilateral hippocampus after CCI and in primary microglia after LPS administration, using hesperidin as a specific inhibitor of Cs. The increase in citrate levels in ipsilateral hippocampus became significant 24 h after injury (Figure 1A). LPS stimulation significantly increased the citrate level in primary microglia, which was significantly reduced after hesperidin pretreatment and became insensitive to LPS (Figure 1B).

In order to confirm the metabolic reprogramming in primary microglia during neuroinflammation, we measured glycolysis by analyzing the extracellular acidification rate (ECAR) and mitochondrial oxidative phosphorylation on the basis of the oxygen consumption rate (OCR). We found that basal respiration and spare maximal respiration were both significantly decreased after LPS simulation. Hesperidin pretreatment could significantly reduce basal respiration and spare maximal respiration in control groups, but there were no significant differences in LPS groups (Figure 1C–E). Furthermore, we found that hesperidin could significantly enhance glycolysis and glycolytic capacity of primary microglia in control groups, which may be due to hesperidin blocking the downstream TCA cycle to a certain extent. But hesperidin could not further increase glycolysis and glycolytic capacity in LPS groups (Figure 1F–H).

**FIGURE 2:**
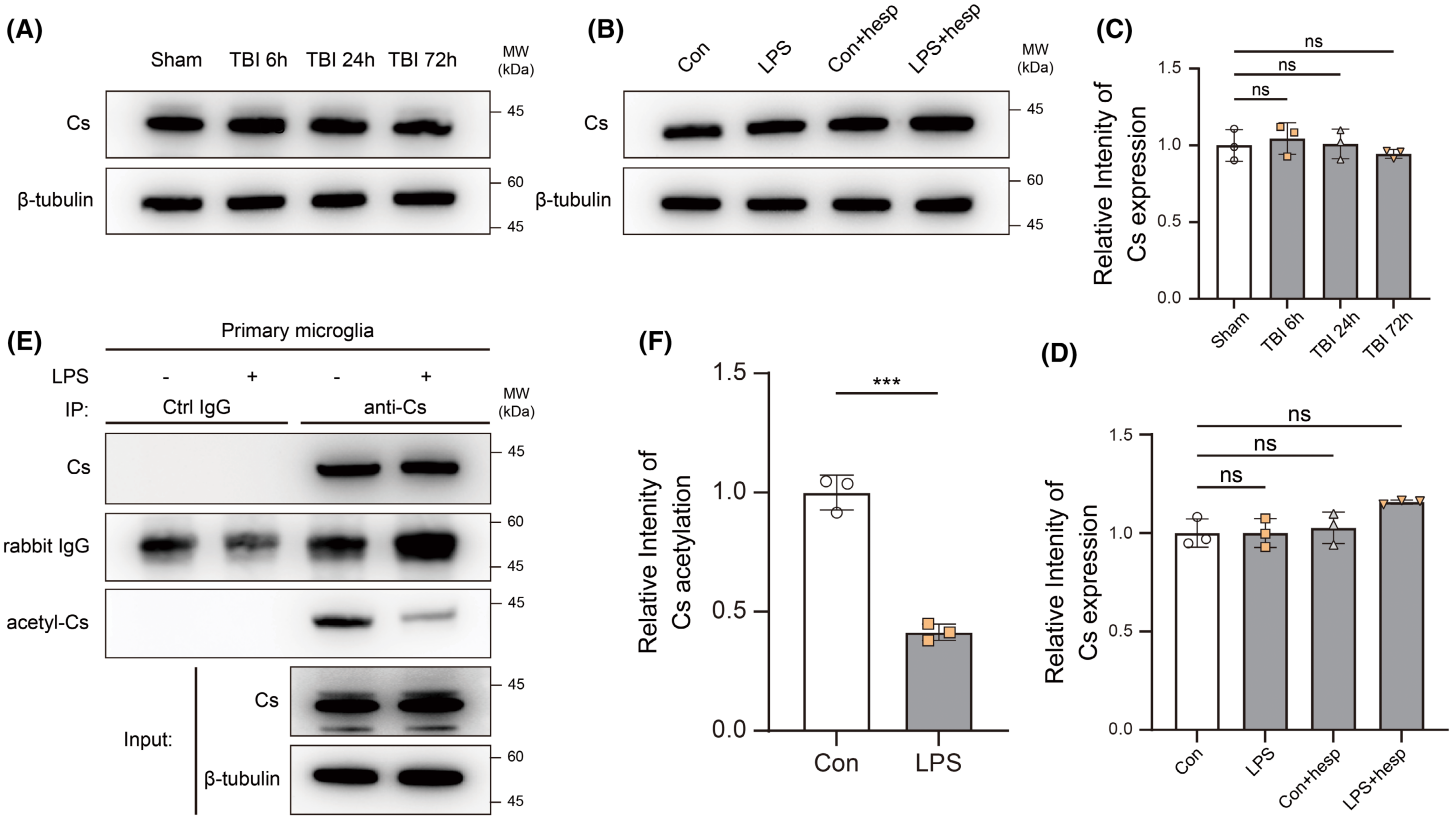
Expression and acetylation of citrate synthase. (A, C) Cs expression in ipsilateral hippocampus 6, 24, and 72 h after traumatic brain injury were analyzed by western blotting (*n* = 3). One‐way ANOVA and Tukey's multiple comparisons test were used. (B, D) Cs expression in primary microglia after LPS stimulation with/without hesperidin pretreatment were analyzed by western blotting (*n* = 3). One‐way ANOVA and Tukey's multiple comparisons test were used. (E, F) Cs acetylation in primary microglia after LPS stimulation with/without hesperidin pretreatment were analyzed by immunoprecipitation and western blotting (*n* = 3). Two‐tailed unpaired *t*‐test was used. Bar graphs are represented as mean ± SD. ***p < 0.001; hesp, hesperidin; ns, no significance.

Since Cs is the indispensable enzyme for citrate synthesis, we detected the changes in Cs expression during neuroinflammation. Our results showed that there was no significant difference in the Cs expression between groups, whether in the ipsilateral hippocampus after injury or in primary microglia stimulated by LPS (Figure 2A–D). Interestingly, acetylation of Cs significantly decreased in primary microglia after LPS administration (Figure 2E,F). The uncropped and unedited blot images are provided in Figure S1.

It could be seen from our data that increased citrate levels in microglia during neuroinflammation may be related to reduced Cs acetylation levels rather than the changes in Cs expression. Hesperidin could reduce citrate in microglia during neuroinflammation but had little effect on microglial metabolic reprogramming after LPS stimulation.

### 2.2 Reduction of Cs K215 acetylation contributes to enhanced enzymatic activity and accumulated citrate

**FIGURE 3:**
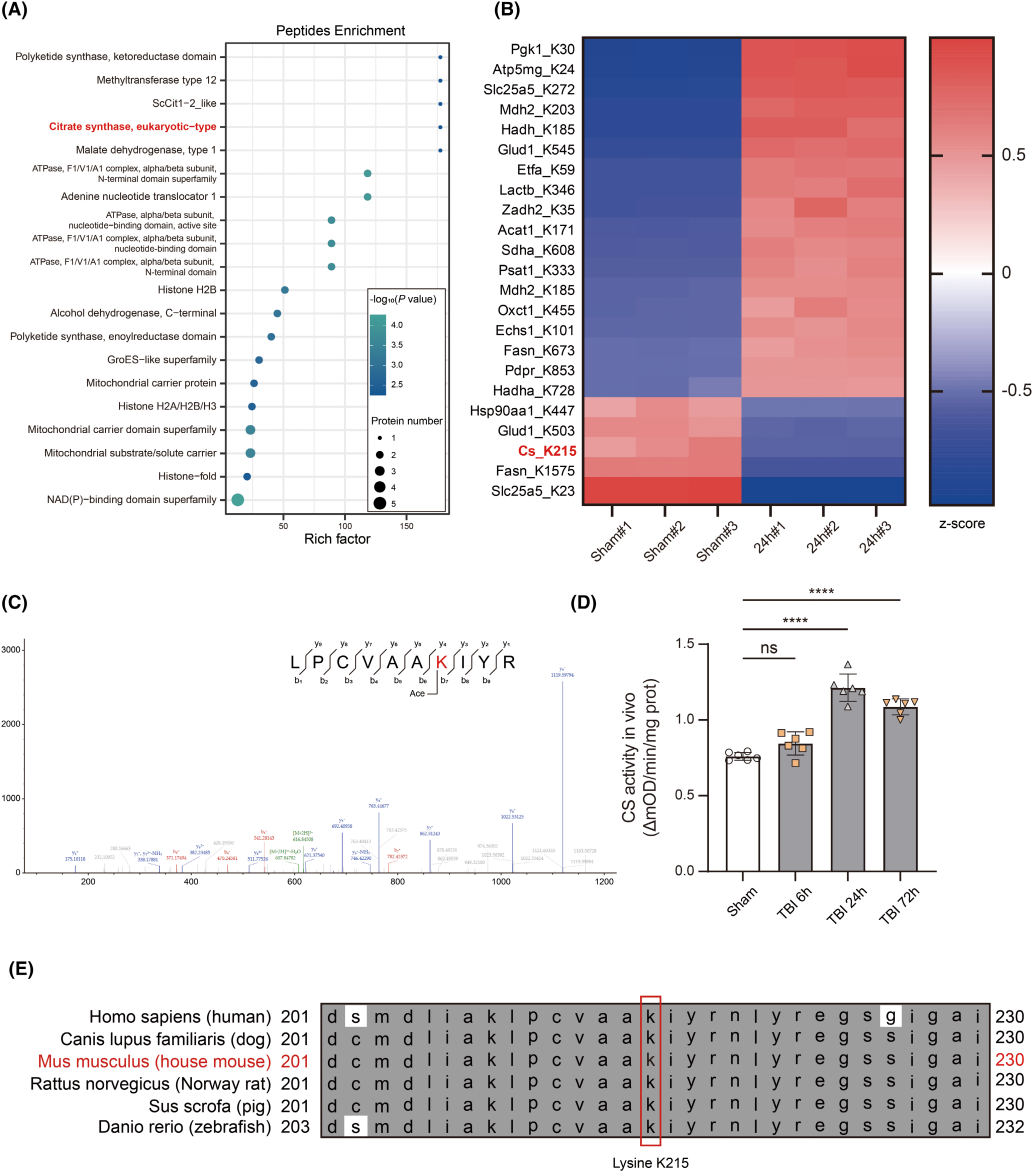
Label‐free acetylation mass spectrometry and bioinformatics. (A) Bubble graph shows that 20 proteins, in which there are significant differences in peptides enrichment, are sorted by rich factor in descending order. Citrate synthase of eukaryotic type is highlighted in red. (B) Heatmap shows the top 23 proteins with significant differences of acetylation modification in ipsilateral hippocampus 24 h after TBI. *p* Value < 0.05 AND |log2(Fold Change)| > 1. Cs K215 is highlighted in red. (C) Acetylated Cs peptide identified by mass spectrometry. Enriched Cs protein was digested by trypsin and further subjected to LC–MS/MS for identification. The lysine conjugated with Ace indicates the acetylation site. (D) Cs enzyme activity in ipsilateral hippocampus 6, 24, and 72 h after traumatic brain injury (*n* = 6). One‐way ANOVA and Tukey's multiple comparisons test were used. (E) Sequence alignment of the region surrounding acetylated K215 among human beings and other vertebrate species. Acetylated lysine is highlighted in red rectangle. Bar graphs are represented as mean ± SD. ****p < 0.0001; ns, no significance.

To investigate the detailed acetylation modification during neuroinflammation, we used label‐free mass spectrometry analysis for protein acetylation modification to detect which proteins and which sites had significant changes in acetylation modification in the injured ipsilateral hippocampus 24 h after TBI. Citrate synthase of eukaryotic type showed up in acetylated peptides enrichment analysis (Figure 3A). The top 23 proteins with the most significant differences and their modification sites were displayed (Figure 3B). The acetylation at K215 on Cs was confirmed (Figure 3C). We assumed that the reduction of Cs K215 acetylation was related with its enhanced enzymatic activity because we found that Cs enzyme activity in ipsilateral hippocampus after TBI significantly increased and peaked at 24 h after injury (Figure 3D). We further performed a cross‐species alignment of the amino acid sequences near Cs K215. It is possible that K215 is a crucial lysine residue since K215 keeps high conservation among various mammals including humans (Figure 3E).

**FIGURE 4:**
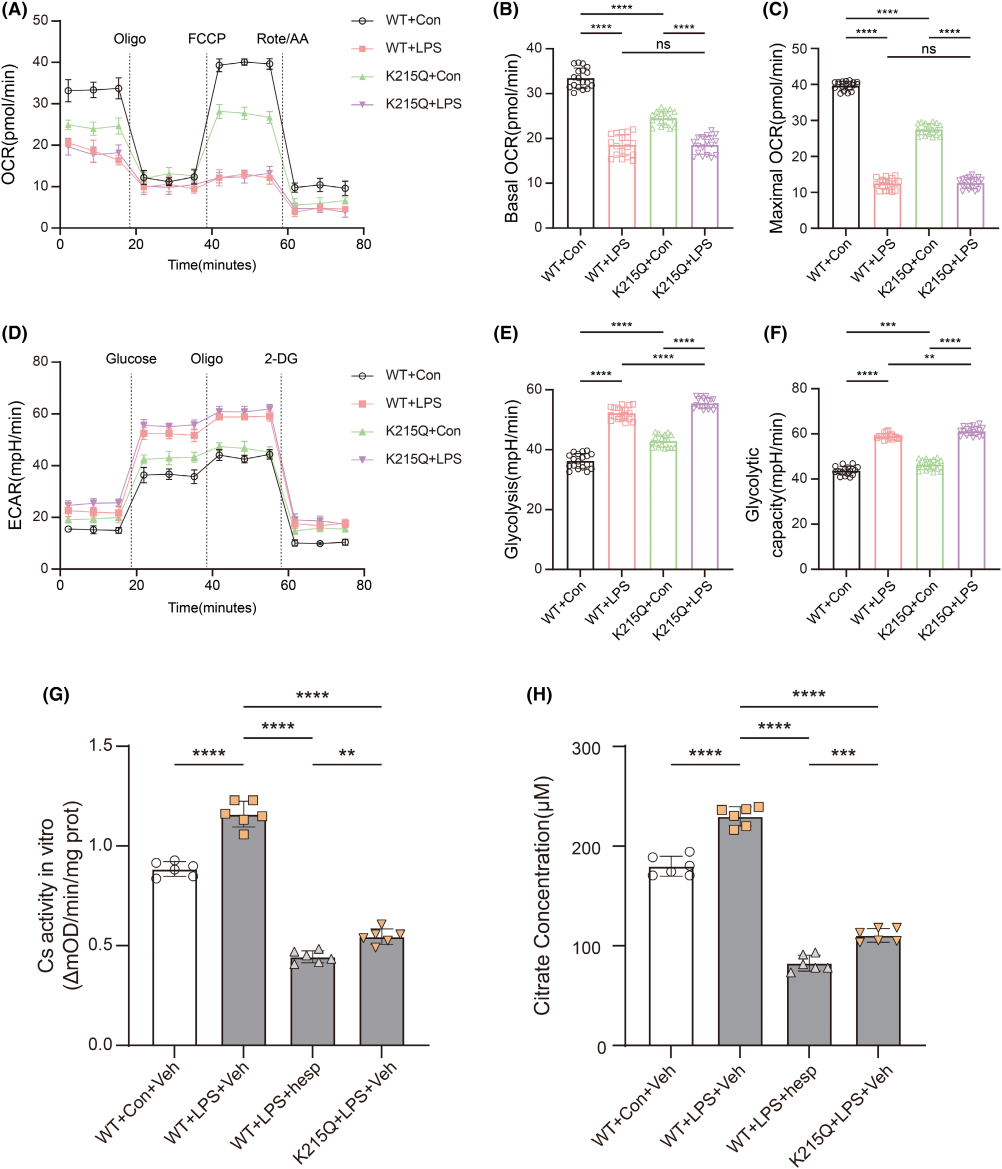
The effects of K215Q mutation on microglial metabolism, Cs activity and citrate accumulation. (A) OCR measurements of primary microglia expressing wildtype Cs or Cs K215Q mutant after LPS stimulation (*n* = 6). (B, C) Statistics on basic OCR and maximum OCR (*n* = 6). One‐way ANOVA and Tukey's multiple comparisons test were used. (D) ECAR measurements of primary microglia expressing wildtype Cs or Cs K215Q mutant after LPS stimulation (*n* = 6). (E, F) Statistics on basic Glycolysis and Glycolytic capacity (*n* = 6). One‐way ANOVA and Tukey's multiple comparisons test were used. (G) Cs enzyme activity in primary microglia expressing Cs wildtype or Cs K215Q mutant after LPS stimulation with/without hesperidin pretreatment (*n* = 6). One‐way ANOVA and Tukey's multiple comparisons test were used. (H) Citrate levels in primary microglia expressing Cs wildtype or Cs K215Q mutant after LPS stimulation with/without hesperidin pretreatment (*n* = 6). One‐way ANOVA and Tukey's multiple comparisons test were used. Bar graphs are represented as mean ± SD. ****p < 0.0001, ***p < 0.001, **p < 0.005; hesp, hesperidin; ns, no significance; veh, vehicle.

Next, we constructed a Cs expression vector with K215Q point mutation to mimic the hyperacetylated state at K215 and transfected primary microglia with rAAV‐cMG2 virus. OCR analysis revealed that Cs K215Q could reduce basal and spare maximal respiration of primary microglia in control groups but neither of them could be further reduced in LPS groups (Figure 4A–C). ECAR analysis revealed that Cs K215Q could slightly increase microglial glycolysis and glycolytic capacity in both control and LPS groups and the differences were significant (Figure 4D–F).

It was shown that LPS could increase the Cs enzyme activity of primary microglia, and Cs K215Q point mutation could exert a significant inhibitory effect on enzyme activity similar to that of hesperidin (Figure 4G). And Cs K215Q could significantly alleviate the citrate accumulation in microglia in LPS groups (Figure 4H).

In this part we proved that Cs K215 hypoacetylation was responsible for its enhanced enzyme activity. Cs K215Q mutation could inhibit its activity and had a slight impact on the metabolic reprogramming of microglia.

### 2.3 Sirt3 is responsible for Cs deacetylation in primary microglia during neuroinflammation

**FIGURE 5:**
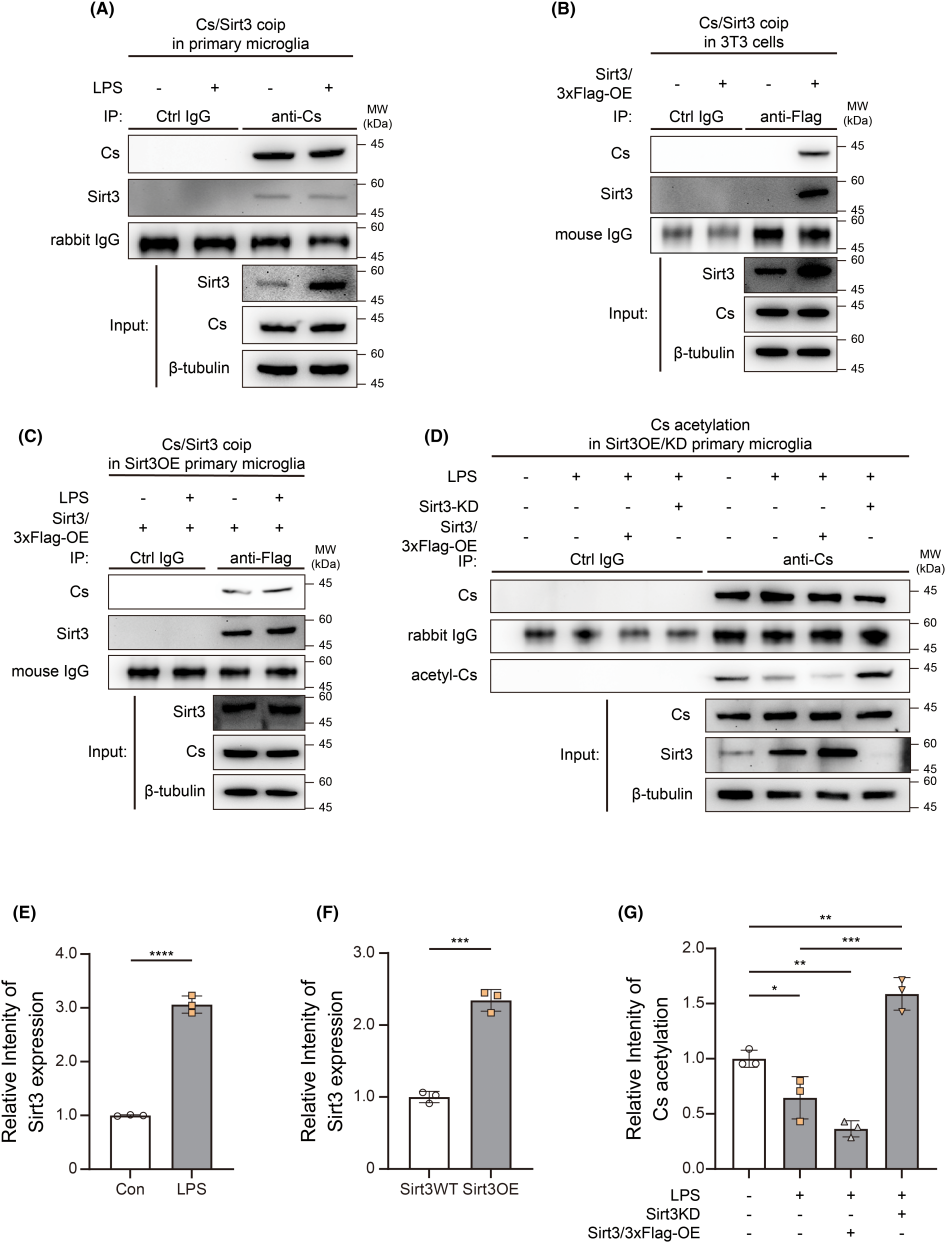
Sirt3 is responsible for Cs deacetylation in primary microglia during neuroinflammation. (A, E) Protein interaction between Cs and Sirt3, and Sirt3 expression were analyzed by immunoprecipitation and western blotting in primary microglia expressing wildtype Cs after LPS stimulation (*n* = 3). Two‐tailed unpaired *t*‐test was used. (B, F) Protein interaction between Cs and Sirt3, and Sirt3 expression were analyzed by immunoprecipitation and western blotting in NIH/3 T3 cells overexpressing Sirt3‐3xFlag (*n* = 3). (C) Protein interaction between Cs and Sirt3 was analyzed by immunoprecipitation and western blotting in primary microglia overexpressing Sirt3‐3xFlag (*n* = 3). Two‐tailed unpaired *t*‐test was used. (D, G) Cs acetylation in primary microglia with wildtype Sirt3, Sirt3 knockdown, or Sirt3‐3xFlag overexpression after LPS stimulation was analyzed by immunoprecipitation and western blotting (*n* = 3). One‐way ANOVA and Tukey's multiple comparisons test were used. Bar graphs are represented as mean ± SD. ****p < 0.0001, ***p < 0.001, **p < 0.005, *p < 0.05; KD, knockdown; ns, no significance; OE: overexpression.

To examine how acetylation modification of microglial Cs K215 is regulated, we measured the expression of the potential responsible enzyme Sirt3, which was regarded as an important deacetylase in mitochondria according to previous studies. The expressions of Sirt3 in primary microglia significantly increased after LPS stimulation (Figure 5A,E), these results are logically consistent with the hypoacetylation of Cs. Moreover, our co‐immunoprecipitation experiments showed that Sirt3 was able to interact with Cs in primary microglia (Figure 5A). To confirm this, we over‐expressed 3xFlag‐tagged Sirt3 in NIH/3 T3 cells and performed co‐immunoprecipitation experiments using an anti‐Flag antibody. This overexpression would not affect the expression baseline of Cs, but it could be seen that Cs interacted with overexpressed Sirt3 (Figure 5B,F). Furthermore, we overexpressed 3xFlag‐tagged Sirt3 in primary microglia with the help of rAAV‐cMG2 and performed co‐immunoprecipitation experiments, obvious co‐precipitation bands of Sirt3 and Cs could be observed regardless of whether microglia were treated by LPS (Figure 5C).

Alterations in Sirt3 expression actually affected the level of Cs acetylation. We compared the level of Cs acetylation under the conditions of Sirt3 wild type, Sirt3 knockdown, and Sirt3 overexpression, respectively. After LPS stimulation, Sirt3 overexpression could significantly reduce Cs acetylation to a very low level; while Cs acetylation could be significantly increased by Sirt3 knockdown, even higher than that in microglia expressing wildtype Sirt3 in control groups (Figure 5D,G). Sirt3 may play an important role in the regulation of acetylation of Cs in microglia during neuroinflammation. The uncropped and unedited blot images are provided in Figure S1.

### 2.4 Cs K215 hypoacetylation could regulate microglial pro‐inflammatory functions independent of Sirt3 expression

**FIGURE 6:**
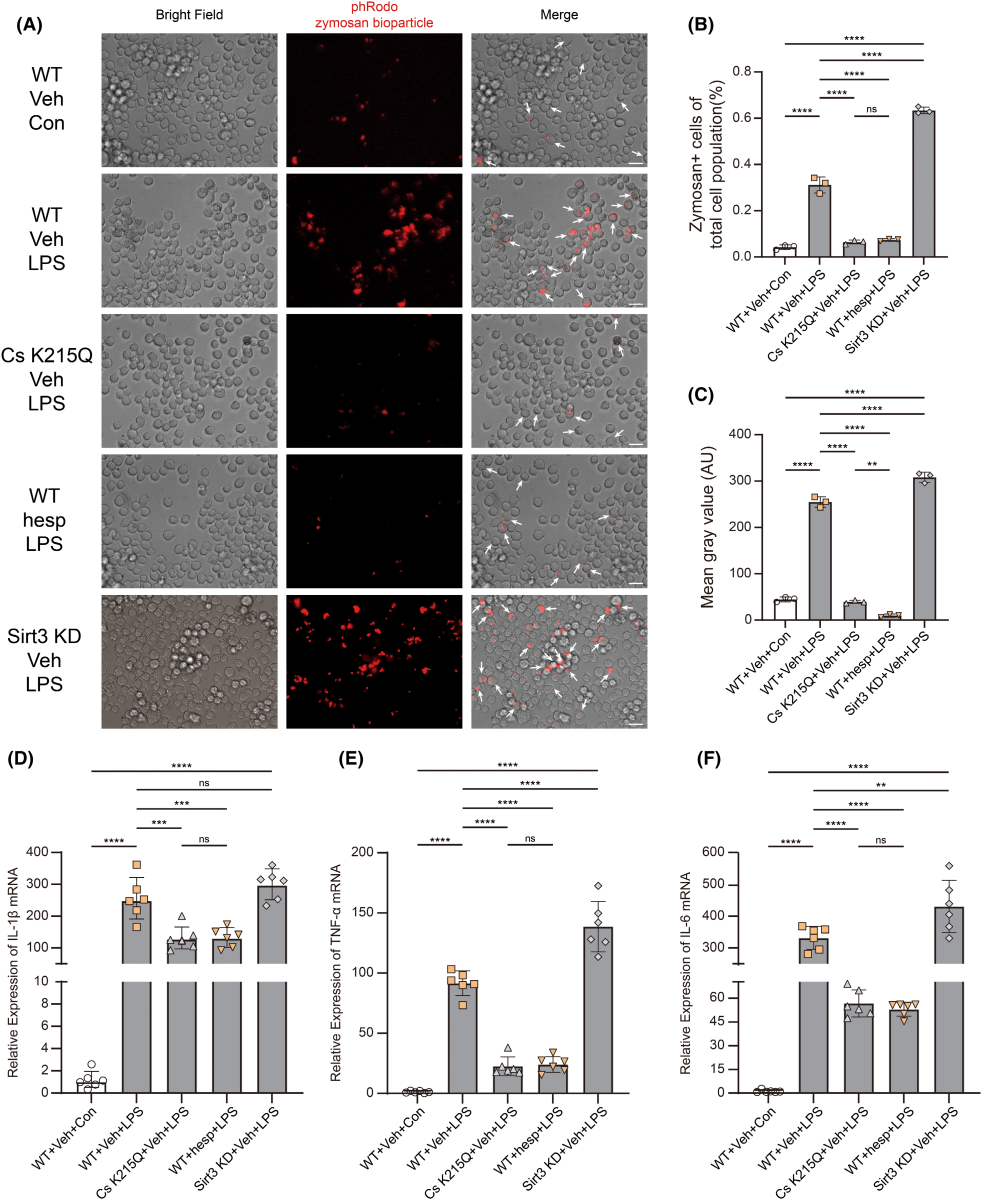
Cs K215 hypoacetylation could regulate microglial pro‐inflammatory functions independent of Sirt3 expression. (A) Representative immunofluorescence and bright field images indicate primary microglial phagocytosis expressing Cs WT, Cs K215Q mutant or Sirt3 knockdown, after LPS and hesperidin administration or not (*n* = 6). Arrows indicate pHRodo Zymosan Bioparticles engulfed by primary microglia. Scale bar: 20 μm. (B) The percentage of pHRodo Zymosan+ microglia in total microglia expressing Cs WT, Cs K215Q mutant, or Sirt3 knockdown, after LPS and hesperidin administration or not (*n* = 6). One‐way ANOVA and Tukey's multiple comparisons test were used. (C) The mean gray value according to the red channel in was calculated by ImageJ (*n* = 6). One‐way ANOVA and Tukey's multiple comparisons test were used. (D–F) IL‐1β, TNF‐α, and IL‐6 mRNA qRT‐PCR is used to evaluate microglial pro‐inflammatory cytokines (*n* = 6). One‐way ANOVA and Tukey's multiple comparisons test were used. Bar graphs are represented as mean ± SD. ****p < 0.0001, ***p < 0.001, **p < 0.005; hesp, hesperidin; KD, knockdown; ns, no significance; veh, vehicle.

The pro‐inflammatory functions of microglia are usually described with its ability to secrete pro‐inflammatory cytokines and to phagocytose extracellular substances. Here we used phRodo‐labeled zymosan bioparticles to measure microglial phagocytosis. LPS stimulation could significantly enhance microglial phagocytosis. Interestingly, Cs K215Q mutation and hesperidin could significantly inhibit microglial phagocytosis while Sirt3 knockdown made microglial phagocytosis even stronger after LPS stimulation (Figure 6A). The proportion of pHRodo‐positive cells and the average red fluorescence intensity statistics confirmed the results (Figure 6B,C). IL‐1β, TNF‐α, and IL‐6, mainly secreted by M1‐activated microglia according to classic neuroinflammation theory, are the most important pro‐inflammatory cytokines involved in cytokine storm in the acute phase after TBI. Both hesperidin and Cs K215Q mutant could significantly reduce the mRNA expression of these three cytokines after LPS stimulation (Figure 6D–F).

It is worth noting that Sirt3 knockdown did not exhibit the expected anti‐inflammatory effects. Sirt3 knockdown caused primary microglia to exhibit significantly stronger phagocytic function and express significantly more pro‐inflammatory cytokines after LPS stimulation than those expressing wild type Sirt3. These results indicates that Sirt3 may have anti‐neuroinflammatory effects through its other more widespread target proteins although Sirt3 is the enzyme responsible for Cs deacetylation.

## 3 DISCUSSION

Hesperidin, a natural phenolic compound, is a flavanone glycoside with various biological effects. Hesperidin is found in tea, olive oil, and especially citrus fruits and is used in traditional Chinese medicine.^19^ It has been shown that hesperidin could play a vital role in antioxidant, anti‐inflammatory, and neuroprotective effects in different models of central nervous system (CNS) disorders.^20^, ^21^, ^22^, ^23^ Previous studies have shown that hesperidin could regulate microglial activation by inhibiting Toll‐like receptor 4 (TLR4) and phosphorylated‐NF‐κB and ameliorate the toxic neurobehavioral effects by reducing oxidative stress and enhancing hippocampal neurogenesis.^24^ However, the effect of hesperidin on microglial metabolism has not received enough attention, although some studies have found that hesperidin may be a potential inhibitor of Cs through molecular docking in silico.^17^ In this study, we successfully inhibited Cs enzyme activity with hesperidin. Hesperidin and Cs K215Q showed similar effects on inhibiting microglial phagocytosis and the expression of inflammatory cytokines, indicating that hesperidin may control neuroinflammation by inhibiting citrate synthesis.

The sirtuins family, including Sirt1‐7, are NAD^+^‐dependent deacetylases with high evolutionary conservation.^25^ Sirt3, Sirt4, and Sirt5 are major, if not exclusively, localized in the mitochondrial matrix.^26^, ^27^, ^28^ Sirt3 is the primary regulator of mitochondrial lysine acetylation,^26^ Sirt5 could regulate lysine malonylation and succinylation,^29^, ^30^ and Sirt4 has weak ADP ribosyltransferase activity.^31^ Previous studies have reported that Sirt3 could regulate the acetylation and enzymatic activity of key enzymes in several metabolic pathways, including complex I and complex II in the respiratory chain,^32^ hydroxymethylglutaryl‐CoA synthase 2 (HMGCS2) in ketogenesis,^33^ superoxide dismutase (SOD2) in oxidative stress,^34^ and so on. Although we have proven in this study that Sirt3 is the enzyme responsible for Cs deacetylation and knockdown of Sirt3 could indeed increase Cs acetylation, Sirt3 knockdown aggravates microglial phagocytosis and pro‐inflammatory cytokine expression. These results are consistent with the anti‐neuroinflammatory effects of Sirt3 reported in previous studies. Therefore, we believe that although Cs K215Q could significantly inhibit pro‐inflammatory activation of microglia, intervention targeting Sirt3 may cause uncontrollable off‐target effects.

The relationship between microglial metabolism and inflammatory function has been a subject of extensive research. Recent studies have shed light on this intricate connection, although certain aspects remain unclear. Emerging evidence suggests that metabolic pathways play a crucial role in regulating microglial activation and subsequent inflammatory responses. Metabolic reprogramming, particularly the shift from oxidative phosphorylation to glycolysis, has been implicated in promoting pro‐inflammatory phenotypes in microglia.^35^ This metabolic switch enhances the production of inflammatory mediators, such as cytokines and reactive oxygen species, contributing to neuroinflammation.^36^ Studies have also highlighted the influence of various metabolic intermediates, including fatty acids, glucose, and ketone bodies, on microglial function. These metabolites can modulate microglial polarization and inflammatory outcomes.^37^ However, several gaps in our understanding persist. It remains unclear how specific metabolic pathways and intermediates precisely regulate microglial inflammatory responses. Additionally, the interplay between microglial metabolism and other factors, such as aging or neurodegenerative diseases, requires further investigation.

Microglial metabolic reprogramming leads to the upregulation and reliance on aerobic glycolysis, which is due to the rate of response required of microglia for an effective neuroinflammation. Glycolysis is able to produce ATP less efficiently, but more rapidly, than OXPHOS. Fragmented TCA cycle is the result of two breakpoints, succinate dehydrogenase (SDH) and isocitrate dehydrogenase (IDH). The accumulation of citrate inside mitochondria could inhibit SDH, which results in further inhibition of the TCA cycle and accumulation of citrate.^38^ In this study, we used hesperidin and Cs K215Q mutation to inhibit Cs enzyme activity, reduce citrate accumulation, and inhibit the pro‐inflammatory functions of microglia. However, microglial metabolic reprogramming has not been reversed, and TCA cycle and OXPHOS have not been improved. The mid‐ to long‐term benefits of this treatment strategy still require further study.

In conclusion, we find that Cs K215 hypoacetylation, regulated by mitochondrial Sirt3, contributes to increased Cs enzyme activity and enhanced microglial pro‐inflammatory functions after TBI in vivo and LPS administration in vitro. Cs K215Q mutation and hesperidin treatment have anti‐inflammatory effects despite microglial metabolic reprogramming remaining.

## 4 METHODS AND MATERIALS

### 4.1 Animals and controlled cortex injury

Adult male C57BL/6J wildtype mice (6–8 weeks old) were used. All animals in this study were approved by the Animal Care and Experimental Committee of the School of Medicine of Shanghai Jiao Tong University. Mice were housed in individual cages in a temperature‐ and humidity‐controlled animal facility with a 12‐h light/dark cycle. Mice were housed in the animal facility for at least 7 days before controlled cortex injury (CCI) surgery, and they were given free access to food and water during this period.

The moderate CCI model was created. After the mice were anesthetized with 4% isoflurane and placed in a stereotactic frame. A circular skull flap in the center of lambda and bregma with a 4 mm diameter was removed. Moderate TBI was then generated by a CCI impactor, with a depth of 2 mm and velocity of 3 m/s, according to a previous protocol. The animals were returned to their cages for recovery after the surgery.

### 4.2 Perfusion and tissue processing

Mice were anesthetized using 4% isoflurane and transcardially perfused with 0.9% NaCl solution. For biochemistry and molecular biological experiments, fresh brain tissues were sampled from ipsilateral cortex and hippocampus and were froze at −80°C for later use.

### 4.3 Primary microglia isolation and culture

Primary microglia were generated by postnatal 0 to 3 days in wildtype (WT) mice. Brains were dissected from postnatal 0–3‐day‐old mice and then dissociated by 0.25% trypsin and trituration until no small clumps were observed in the cell suspension. Single‐cell suspensions were obtained by passing the suspension through a 70‐mm nylon cell strainer. Finally, the cell suspension was plated onto 75‐cm^2^ poly‐lysine‐coated culture flasks. The cells were grown in Dulbecco's modified Eagle medium (DMEM, Invitrogen, Waltham, MA, USA) supplemented with 20% fetal bovine serum (FBS, Gibco, Grand Island, NY, USA) and 1% penicillin‐streptomycin (Invitrogen). After 10 days of culture, primary microglia were separated and collected for experiments.

### 4.4 LPS and hesperidin treatment on primary microglia

To induce metabolic reprogramming, primary microglia were treated for 18 h with 5 μg/mL LPS (from *E. coli*, Sigma, L4391) in DMEM +10% FBS. Controls received vehicle solution (PBS) only. To inhibit Cs activity, primary microglia were pretreated with 50 μM hesperidin (MCE, HY‐15337) or vehicle (saline) in DMEM +10% FBS. After 24 h of hesperidin pretreatment, LPS or LPS‐vehicle solution was used, and cells were co‐treated with hesperidin and LPS for another 18 h.

### 4.5 Cs K215Q mutant construction and transfection

Cs wildtype vector was created by inserting a Cs transcript NM_026444.4 behind the CMV promotor of pcDNA3.1. Cs K215Q mutant vector was created by the similar way but the adenine A834 in cDNA was replaced by guanine G834. These plasmids were transfected into NIH/3 T3 cells with the help of Lipofectamine 3000 agents (ThermoFisher, L3000008).

### 4.6 Sirt3 knockdown and overexpression

Sirt3 knockdown was performed by using short hairpin RNA (shRNA). The following shRNA were used in this study:

shScramble: CAACAAGATGAAGAGCACCAA.

shSIRT3: GGTGGAAGAAGGTCCATATCT.

Sirt3 wildtype overexpression vector was created by inserting a Sirt3 transcript NM_001177804.1, which was followed by 3xFlag, behind the CMV promotor of pcDNA3.1. These plasmids were transfected into NIH/3 T3 cells with the help of Lipofectamine 3000 agents (ThermoFisher, L3000008).

### 4.7 rAAV2‐cMG2 virus packaging

The plasmid of rAAV2‐cMG2 viral capsid is the gift from Prof. PhD. Minmin Luo of Tsinghua Institute of Multidisciplinary Biomedical Research (TIMBR), Beijing, China. The vectors of wildtype Cs, Cs K215Q mutant, Sirt3‐3xFlag overexpression and Sirt3 knockdown were packaged into this virus.

### 4.8 Virus transfection in vitro

rAAV2‐cMG2 was used for primary microglia transfection in vitro. primary microglia were seeded at 5 × 10^5^ cells per well into 6‐well plates and were transfected by the virus at a multiplicity of infection (MOI) of 10,000. After 2 days, these cells could be used for the follow‐up experiments.

### 4.9 Microglial phagocytosis assay

For microglial phagocytosis assays, primary microglia were split into 96‐well plates at 1000 cells per well in DMEM +10% FBS and treated with LPS, hesperidin and/or vehicle solutions as described above. Following specific treatments, 5 ng of pHRodo Red Zymosan Bioparticles (Thermo Fisher Scientific, P35364) in 100 μL of DMEM +5% FBS was added per well. Three bright field and red fluorescent images per well were acquired after 2 h using a fluorescence microscope (Olympus IXplore Standard). To analyze the phagocytosis ability of primary microglia, the numbers of total pHRodo^+^ cells and of total primary microglia were counted, and the percentage of pHRodo^+^ primary microglia was calculated.

### 4.10 Cytokine detection by qRT‐PCR

Total RNA was isolated from primary microglia after specific treatments, by using Trizol (Invitrogen) according to the manufacturer's instructions. cDNA was synthesized by using a reverse transcription kit (TAKARA). PCR was performed by using SYBR Advantage Premix (TAKARA). The following primers for the cytokines which were detected in this study were used:

IL‐1β: Forward: 5′‐GCAACTGTTCCTGAACTCAACT‐3′; Reverse: 5′‐ATCTTTTGGGGTCCGTCAACT‐3′.

TNF‐α: Forward: 5′‐CCCTCACACTCAGATCATCTTCT‐3′; Reverse: 5′‐GCTACGACGTGGGCTACAG ‐3′.

IL‐6: Forward: 5′‐TAGTCCTTCCTACCCCAATTTCC ‐3′; Reverse: 5′‐TTGGTCCTTAGCCACTCCTTC ‐3′.

GAPDH was used as the control:

Forward: 5′‐AGGTCGGTGTGAACGGATTTG‐3′; Reverse: 5′‐TGTAGACCATGTAGTTGAGGTCA‐3′.

All experiments were done in triplicates.

### 4.11 Label‐free mass spectrometry analysis for protein acetylation modification

Sham and 24 h post‐TBI male wildtype mice (*n* = 3 mice per group) were perfused, and the hippocampus were extracted. SDT buffer was added to the sample and transferred to 2‐mL tubes with amount quartz sand. The lysate was homogenized by MP Fastprep‐24 Automated Homogenizer (6.0 M/S, 30s, twice). The homogenate was sonicated and then boiled for 10 min. After centrifuged at 14000*g* for 15 min, the supernatant was filtered with 0.22 μm filters. The filtrate was quantified with the BCA Protein Assay Kit (P0012, Beyotime). The sample was stored at −80°C. 20 μg of proteins for each sample were mixed with 6X loading buffer respectively and boiled for 5 min. The proteins were separated on 12% SDS‐PAGE gel. Protein bands were visualized by Coomassie Blue R‐250 staining.

5 mg of proteins for each sample were reduced with 100 mM DTT for 5 min at 100°C. Then the detergent, DTT and other low‐molecular‐weight components were removed using UA buffer (8 M Urea, 150 mM Tris–HCl pH 8.5) by repeated ultrafiltration (Sartorius, 30 kD). Then 100 μL iodoacetamide (100 mM IAA in UA buffer) was added to block reduced cysteine residues and the samples were incubated for 30 min in darkness. The filters were washed with 100 μL UA buffer three times and then 100 μL 50 mM NH_4_HCO_3_ buffer twice. Finally, the protein suspensions were digested with 4 μg trypsin (Promega) in 40 μL 50 mM NH_4_HCO_3_ buffer overnight at 37°C, and the resulting peptides were collected as a filtrate. The peptide segment was desalted by C18 column. The peptide content was estimated by UV light spectral density at 280 nm using an extinctions coefficient of 1.1 of 0.1% (g/L) solution that was calculated on the basis of the frequency of tryptophan and tyrosine in vertebrate proteins. The peptides mixture was subjected to Acetyl‐Lysine Motif [Ac‐K] Kit (Cell Signaling Technology, 13416S) for Kac enrichment.

Samples were analyzed on a nanoElute (Bruker, Bremen, Germany) coupled to a timsTOF Pro (Bruker, Bremen, Germany) equipped with a CaptiveSpray source. The MS data were analyzed using MaxQuant software version 1.6.17.0. MS data were searched against the database.

### 4.12 Citrate and Cs enzyme activity detection

The frozen brain samples and primary microglia after LPS/vehicle and/or hesperidin administration were used for citrate levels and Cs enzyme activity detection by Citric Acid Content Assay Kit (Solarbio, BC2155) and Citrate Synthase Activity Assay Kit (Solarbio, BC1065). The corresponding users' guide were followed. The data of Cs enzyme activity were normalized by the total protein per sample.

### 4.13 Seahorse extracellular flux assay

The oxygen consumption rate (OCR) and the extracellular acidification rate (ECAR) were performed using the Seahorse XF Cell Mito Stress Test Kit (103015–100, Agilent Technologies) and Seahorse XF Glycolysis Stress Test Kit (103020–100, Agilent Technologies) according to the manufacturer's protocols. 3 × 10^5^ primary microglia per well were seeded onto a Seahorse XF 96‐well culture microplate overnight and treated with LPS, hesperidin, and/or vehicle as described above. Upon measurement, cells were washed twice with XF assay medium (102353–100, Agilent Technologies) and maintained in it. After baseline measurements, oligomycin, carbonyl cyanide‐p‐trifluoromethoxyphenylhydrazone (FCCP) and Rotenone (Rote), and antimycin A (AA) were injected into the wells sequentially at specific time points for OCR analysis. For ECAR analysis, glucose, oligomycin (Oligo), and 2‐DG were injected. The results were normalized to cell number, and data are presented as pmol/min for OCR and mPH/min for ECAR.

### 4.14 Coimmunoprecipitation, acetyl‐lysine detection and western blotting

Primary microglia after LPS/vehicle and/or hesperidin administration were lysed in IP buffer (Solarbio, R0100). The lysates were centrifuged at 12,000*g* for 15 min at 4°C. The protein concentration was estimated by the Bradford method. Parts of the lysates were subjected to immunoprecipitation (IP) using rabbit anti‐Cs antibody (1:50; Abcam, ab96600), mouse anti‐Flag antibody (1:50; Abcam, ab125243), or rabbit control IgG (1:50; Abcam, ab128870). The samples were separated by 8% SDS polyacrylamide gel electrophoresis and electro‐transferred onto a polyvinylidene difluoride membrane (Bio‐Rad Lab, Hercules, CA).

The membrane was blocked with 5% skim milk for 2 h at room temperature and incubated with the following primary antibodies for 12 h at 4°C: rabbit anti‐Cs antibody (1:1000; Abcam, ab96600); rabbit anti‐Sirt3 (1:1000; Abcam, ab189860); HRP‐conjugated rabbit anti‐β‐tubulin (1:10,000; Abcam, ab21058). The level of acetyl‐Cs was detected by mouse anti‐acetyl‐lysine antibody (1:1000; Cell Signaling, #9681). After the membrane had been washed 3 times in a mixture of Tris‐buffered saline and Tween‐20 (TBST) for 10 min each time, it was incubated with the appropriate horseradish peroxidase‐conjugated secondary antibody (1:10,000 dilution in TBST) for 1 h. For better coimmunoprecipitation (coIP) bands detection, a HRP‐conjugated mouse anti‐rabbit IgG light chain (1:10,000; Abbkine, A25022) was used. The blotted protein bands were visualized and analyzed by ChemiDoc system and ImageLab software from Bio Rad.

### 4.15 Statistical analysis

All data are presented as the mean ± the standard deviation (SD). Graphpad Prism 9.4.0 for Windows was used for statistical analysis of the data. All data were first subjected to Shapiro‐Wilk or Kolmogorov‐Smirnov test for normality, and then one‐way analysis of variance and post hoc comparisons were made with Tukey or two‐tailed *t*‐test as recommended by the software. Data that do not exhibit a normal distribution were analyzed via a nonparametric equivalent based on Kruskal‐Wallis test and Dunn's multiple comparisons. The specific statistical methods used in each experiment are listed in Figure Legends. Statistical significance was inferred at *p* < 0.05.

## AUTHOR CONTRIBUTIONS

Fengchen Zhang and Tao Lv contributed equally to this study as first authors. They were involved in the design and data collection, interpretation of the results, and writing of the manuscript. Jie Li, Jie Lian, and Hui Wu were involved in data analysis and interpretation. They also provided critical insights and feedback during the manuscript writing process. Yichao Jin, Feng Jia, and Xiaohua Zhang contributed equally as corresponding authors. They provided overall supervision and guidance throughout the study. They also provided intellectual input and expertise in the field of neurobiology. All authors reviewed and approved the final version of the manuscript for publication.

## CONFLICT OF INTEREST STATEMENT

All authors declare that no conflicts of interest exist.

## Supporting information


Figure S1

## Data Availability

All study data are available from the corresponding author by e‐mail.

## References

[1] Prinz M , Jung S , Priller J . Microglia biology: one century of evolving concepts. Cell. 2019;179(2):292‐311. 31585077 10.1016/j.cell.2019.08.053

[2] Geric I , Schoors S , Claes C , et al. Metabolic reprogramming during microglia activation. Immunometabolism. 2019;1:1.

[3] Williams NC , O'Neill LA . A role for the Krebs cycle intermediate citrate in metabolic reprogramming in innate immunity and inflammation. Front Immunol. 2018;9:141. 29459863 10.3389/fimmu.2018.00141 PMC5807345

[4] Li Y , Li Y‐C , Liu X‐T , et al. Blockage of citrate export prevents TCA cycle fragmentation via Irg1 inactivation. Cell Rep. 2022;38(7):110391. 35172156 10.1016/j.celrep.2022.110391

[5] Infantino V , Convertini P , Cucci L , et al. The mitochondrial citrate carrier: a new player in inflammation. Biochem J. 2011;438(3):433‐436. 21787310 10.1042/BJ20111275

[6] Infantino V , Iacobazzi V , Menga A , Avantaggiati ML , Palmieri F . A key role of the mitochondrial citrate carrier (SLC25A1) in TNFα‐and IFNγ‐triggered inflammation. Biochim Biophys Acta. 2014;1839(11):1217‐1225. 25072865 10.1016/j.bbagrm.2014.07.013 PMC4346166

[7] Infantino V , Iacobazzi V , Palmieri F , Menga A . ATP‐citrate lyase is essential for macrophage inflammatory response. Biochem Biophys Res Commun. 2013;440(1):105‐111. 24051091 10.1016/j.bbrc.2013.09.037

[8] Wellen KE , Hatzivassiliou G , Sachdeva UM , Bui TV , Cross JR , Thompson CB . ATP‐citrate lyase links cellular metabolism to histone acetylation. Science. 2009;324(5930):1076‐1080. 19461003 10.1126/science.1164097 PMC2746744

[9] Cherry JD , Olschowka JA , O'Banion MK . Neuroinflammation and M2 microglia: the good, the bad, and the inflamed. J Neuroinflammation. 2014;11(1):1‐15. 24889886 10.1186/1742-2094-11-98 PMC4060849

[10] Koronowski KB , Khoury N , Saul I , et al. Neuronal SIRT1 (silent information regulator 2 homologue 1) regulates glycolysis and mediates resveratrol‐induced ischemic tolerance. Stroke. 2017;48(11):3117‐3125. 29018134 10.1161/STROKEAHA.117.018562 PMC5654689

[11] He W , Li Q , Li X . Acetyl‐CoA regulates lipid metabolism and histone acetylation modification in cancer. Biochim Biophys Acta. 2023;1878(1):188837. 10.1016/j.bbcan.2022.188837 36403921

[12] Xiong H , Wang J , Ran Q , et al. Hesperidin: a therapeutic agent for obesity. Drug des Devel Ther. 2019;13:3855‐3866. 10.2147/DDDT.S227499 PMC6859214 32009777

[13] Choi S‐S , Lee S‐H , Lee K‐A . A comparative study of hesperetin, hesperidin and hesperidin glucoside: antioxidant, anti‐inflammatory, and antibacterial activities in vitro. Antioxidants. 2022;11(8):1618. 36009336 10.3390/antiox11081618 PMC9405481

[14] Guazelli CF , Fattori V , Ferraz CR , et al. Antioxidant and anti‐inflammatory effects of hesperidin methyl chalcone in experimental ulcerative colitis. Chem Biol Interact. 2021;333:109315. 33171134 10.1016/j.cbi.2020.109315

[15] Pandey P , Khan F . A mechanistic review of the anticancer potential of hesperidin, a natural flavonoid from citrus fruits. Nutr Res. 2021;92:21‐31. 34273640 10.1016/j.nutres.2021.05.011

[16] Ahmad S , Mittal S , Gulia R , et al. Therapeutic role of hesperidin in collagen‐induced rheumatoid arthritis through antiglycation and antioxidant activities. Cell Biochem Funct. 2022;40(5):473‐480. 35657316 10.1002/cbf.3708

[17] Rao A , Haque S , El‐Enshasy HA , Singh V , Mishra BN . RSM–GA based optimization of bacterial PHA production and in Silico modulation of citrate synthase for enhancing PHA production. Biomolecules. 2019;9(12):872. 31842491 10.3390/biom9120872 PMC6995514

[18] Lauro C , Chece G , Monaco L , et al. Fractalkine modulates microglia metabolism in brain ischemia. Front Cell Neurosci. 2019;13:414. 31607865 10.3389/fncel.2019.00414 PMC6755341

[19] Musa AE , Omyan G , Esmaely F , Shabeeb D . Radioprotective effect of hesperidin: a systematic review. Medicina. 2019;55(7):370. 31336963 10.3390/medicina55070370 PMC6681345

[20] Ikram M , Muhammad T , Rehman SU , et al. Hesperetin confers neuroprotection by regulating Nrf2/TLR4/NF‐κB signaling in an Aβ mouse model. Mol Neurobiol. 2019;56:6293‐6309. 30756299 10.1007/s12035-019-1512-7

[21] Muhammad T , Ikram M , Ullah R , Rehman SU , Kim MO . Hesperetin, a citrus flavonoid, attenuates LPS‐induced neuroinflammation, apoptosis and memory impairments by modulating TLR4/NF‐κB signaling. Nutrients. 2019;11(3):648. 30884890 10.3390/nu11030648 PMC6471991

[22] Antunes MS , Cattelan Souza L , Ladd FVL , et al. Hesperidin ameliorates anxiety‐depressive‐like behavior in 6‐OHDA model of Parkinson's disease by regulating striatal cytokine and neurotrophic factors levels and dopaminergic innervation loss in the striatum of mice. Mol Neurobiol. 2020;57:3027‐3041. 32458386 10.1007/s12035-020-01940-3

[23] Zhu X , Liu H , Liu Y , et al. The antidepressant‐like effects of hesperidin in streptozotocin‐induced diabetic rats by activating Nrf2/ARE/glyoxalase 1 pathway. Front Pharmacol. 2020;11:1325. 32982741 10.3389/fphar.2020.01325 PMC7485173

[24] Noshy PA , Azouz RA . Neuroprotective effect of hesperidin against emamectin benzoate‐induced neurobehavioral toxicity in rats. Neurotoxicol Teratol. 2021;86:106981. 33838246 10.1016/j.ntt.2021.106981

[25] Ji Z , Liu G‐H , Qu J . Mitochondrial sirtuins, metabolism, and aging. J Genet Genomics. 2022;49(4):287‐298. 34856390 10.1016/j.jgg.2021.11.005

[26] Dikalova AE , Pandey A , Xiao L , et al. Mitochondrial deacetylase Sirt3 reduces vascular dysfunction and hypertension while Sirt3 depletion in essential hypertension is linked to vascular inflammation and oxidative stress. Circ Res. 2020;126(4):439‐452. 31852393 10.1161/CIRCRESAHA.119.315767 PMC7035170

[27] Zaganjor E , Yoon H , Spinelli JB , et al. SIRT4 is an early regulator of branched‐chain amino acid catabolism that promotes adipogenesis. Cell Rep. 2021;36(2):109345. 34260923 10.1016/j.celrep.2021.109345 PMC8320302

[28] Liu X , Rong F , Tang J , et al. Repression of p53 function by SIRT5‐mediated desuccinylation at lysine 120 in response to DNA damage. Cell Death Differ. 2022;29(4):722‐736. 34642466 10.1038/s41418-021-00886-w PMC8989948

[29] Du J , Zhou Y , Su X , et al. Sirt5 is a NAD‐dependent protein lysine demalonylase and desuccinylase. Science. 2011;334(6057):806‐809. 22076378 10.1126/science.1207861 PMC3217313

[30] Xiao Z‐P , Lv T , Hou P‐P , et al. Sirtuin 5‐mediated lysine desuccinylation protects mitochondrial metabolism following subarachnoid hemorrhage in mice. Stroke. 2021;52(12):4043‐4053. 34807744 10.1161/STROKEAHA.121.034850

[31] Haigis MC , Mostoslavsky R , Haigis KM , et al. SIRT4 inhibits glutamate dehydrogenase and opposes the effects of calorie restriction in pancreatic β cells. Cell. 2006;126(5):941‐954. 16959573 10.1016/j.cell.2006.06.057

[32] Ahn BH . A role for the mitochondrial deacetylase Sirt3 in regulating energy homeostasis. Proc Natl Acad Sci USA. 2008;105:14447‐14452. 18794531 10.1073/pnas.0803790105 PMC2567183

[33] Shimazu T , Hirschey MD , Hua L , et al. SIRT3 deacetylates mitochondrial 3‐hydroxy‐3‐methylglutaryl CoA synthase 2 and regulates ketone body production. Cell Metab. 2010;12(6):654‐661. 21109197 10.1016/j.cmet.2010.11.003 PMC3310379

[34] Qiu X , Brown K , Hirschey MD , Verdin E , Chen D . Calorie restriction reduces oxidative stress by SIRT3‐mediated SOD2 activation. Cell Metab. 2010;12(6):662‐667. 21109198 10.1016/j.cmet.2010.11.015

[35] Wang Q , Lu M , Zhu X , et al. The role of microglia immunometabolism in neurodegeneration: focus on molecular determinants and metabolic intermediates of metabolic reprogramming. Biomed Pharmacother. 2022;153:113412. 36076537 10.1016/j.biopha.2022.113412

[36] Yu H , Chang Q , Sun T , et al. Metabolic reprogramming and polarization of microglia in Parkinson's disease: role of inflammasome and iron. Ageing Res Rev. 2023;90:102032. 37572760 10.1016/j.arr.2023.102032

[37] Bernier L‐P , York EM , MacVicar BA . Immunometabolism in the brain: how metabolism shapes microglial function. Trends Neurosci. 2020;43(11):854‐869. 32958333 10.1016/j.tins.2020.08.008

[38] Hillar M , Lott V , Lennox B . Correlation of the effects of citric acid cycle metabolites on succinate oxidation by rat liver mitochondria and submitochondrial particles. J Bioenerg. 1975;7(1):1‐16. 1176438 10.1007/BF01558459

