# Supplementary material for: Citrate synthase lysine K215 hypoacetylation contributes to microglial citrate accumulation and pro‐inflammatory functions after traumatic brain injury
Source: CNS Neurosci Ther. 2024 Feb 8;30(2):e14567. doi: 10.1111/cns.14567 (PMC10851320; doi:10.1111/cns.14567)
Supplement: Supplementary file 1 — Figure S1 [file CNS-30-e14567-s001.pdf]

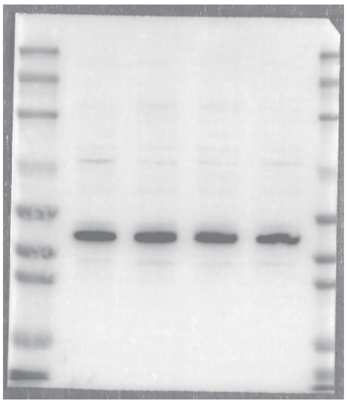

Cs after TBI in vivo

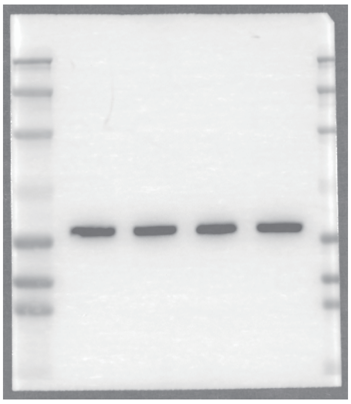

β-tubulin after TBI in vivo

unedited images for Figure 2a

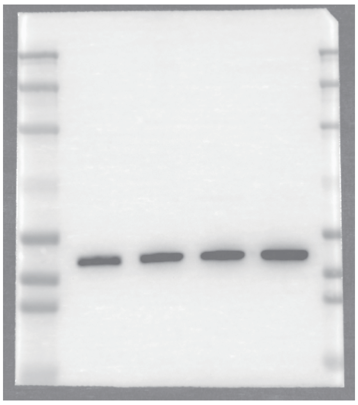

Cs in vitro

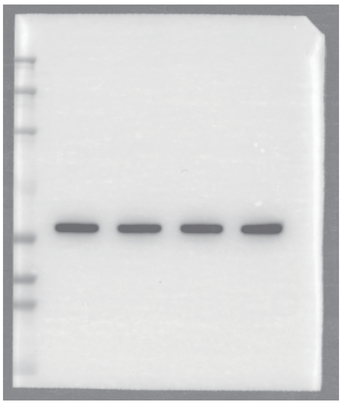

β-tubulin in vitro

unedited images for Figure 2b

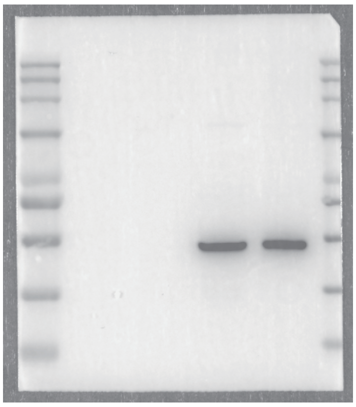

anti-Cs IP
anti-Cs IB

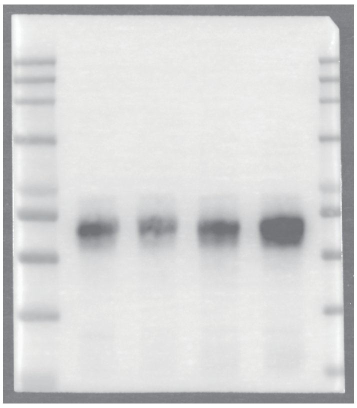

rabbit IgG

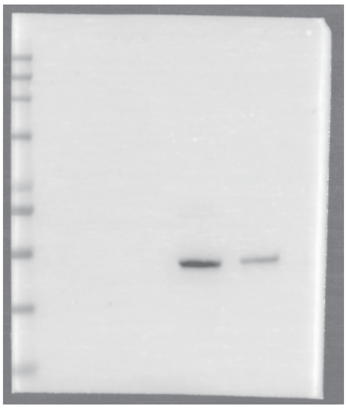

anti-Cs IP
anti-Acetylated-Lysine IB

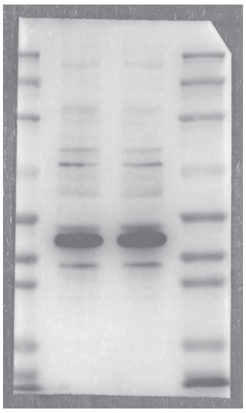

Cs Input

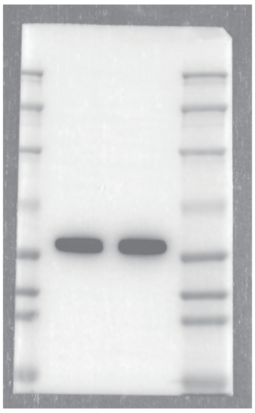

β-tubulin Input

unedited images for Figure 2e

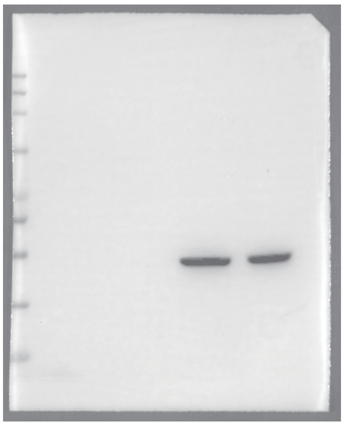

anti-Cs IP
anti-Cs IB

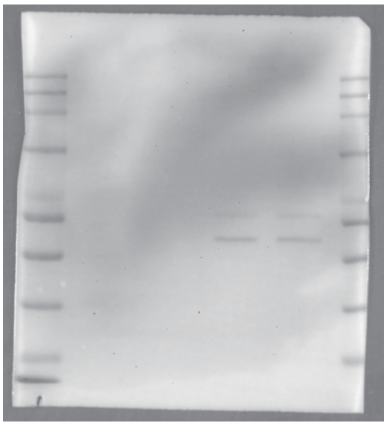

anti-Cs IP
anti-Sirt3 IB

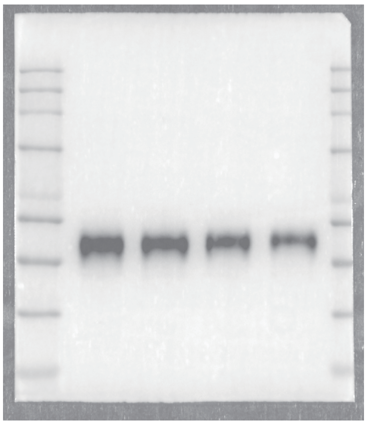

rabbit IgG

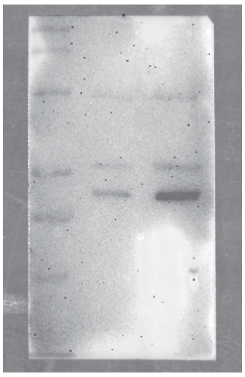

Sirt3 Input

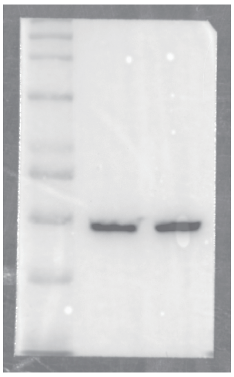

Cs Input

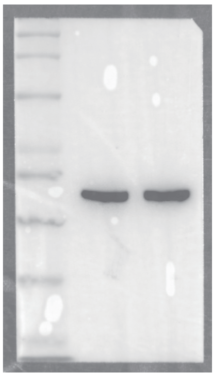

β-tubulin Input

unedited images for Figure 5a

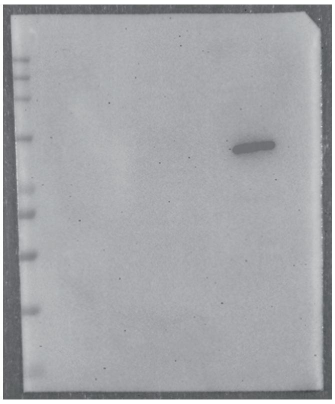

anti-Flag IP
anti-Sirt3 IB

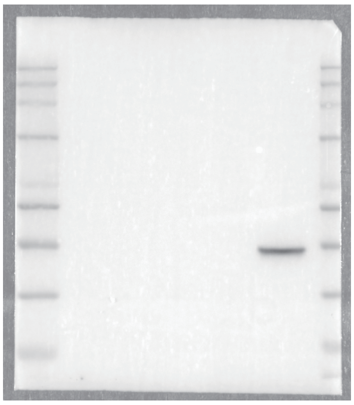

anti-Flag IP
anti-Cs IB

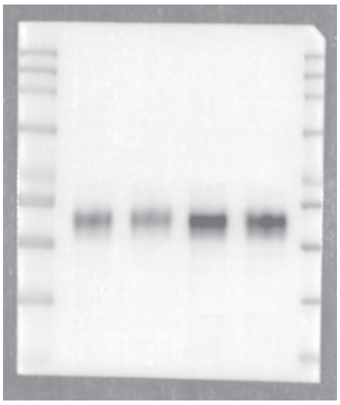

mouse IgG

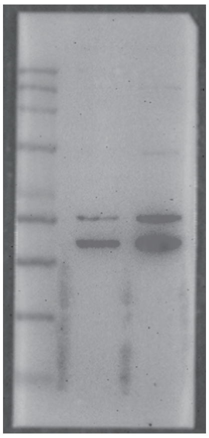

Sirt3 Input

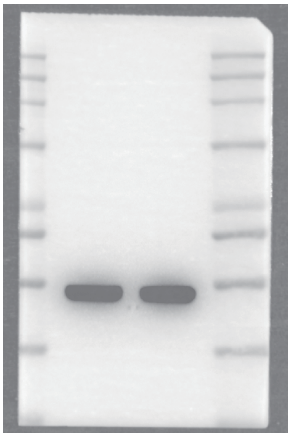

Cs Input

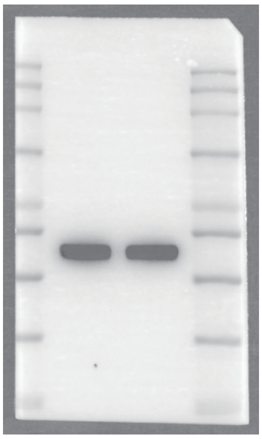

β-tubulin Input

unedited images for Figure 5b

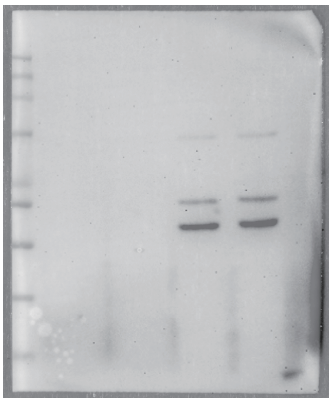

anti-Flag IP
anti-Sirt3 IB

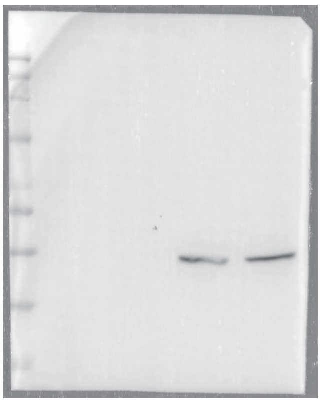

anti-Flag IP
anti-Cs IB

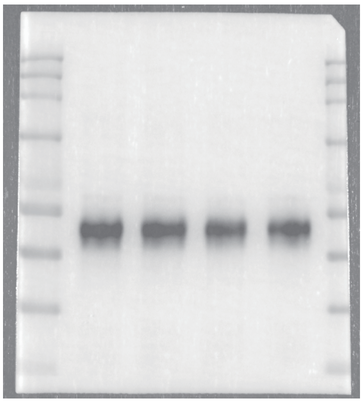

mouse IgG

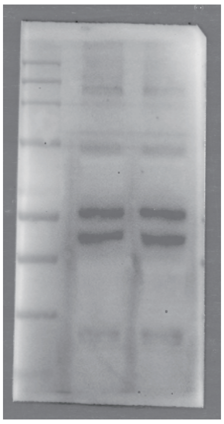

Sirt3 Input

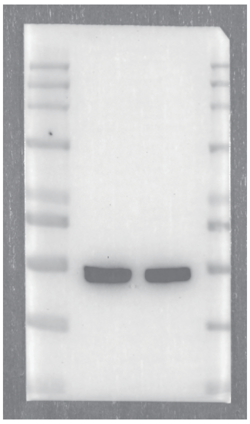

Cs Input

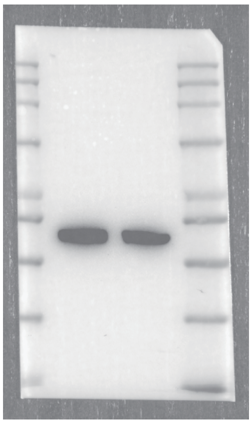

β-tubulin Input

unedited images for Figure 5c

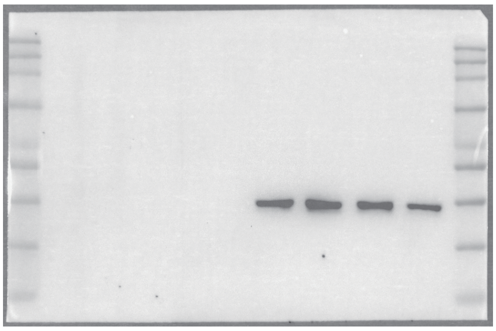

anti-Cs IP
anti-Cs IB

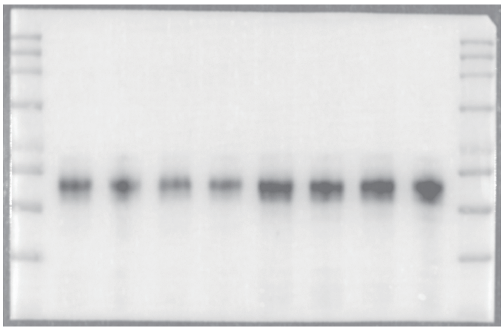

rabbit IgG

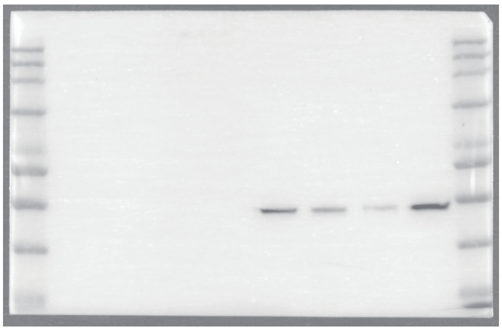

anti-Cs IP
anti-Acetylated-Lysine IB

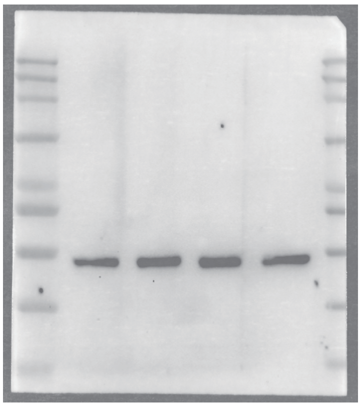

Cs Input

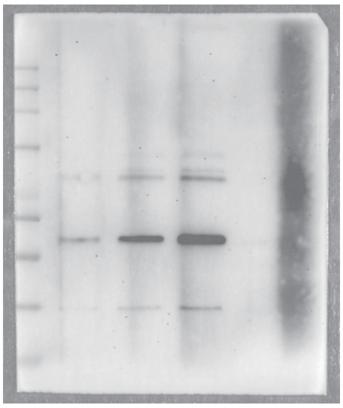

Sirt3 Input

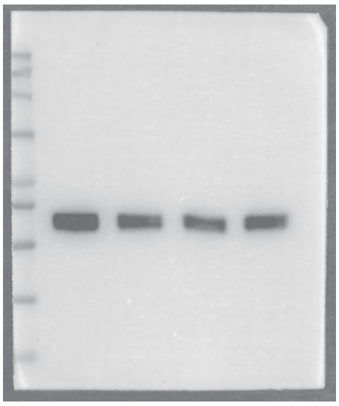

β-tubulin Input

unedited images for Figure 5d
